# Corni Fructus Containing Formulation Attenuates Weight Gain in Mice with Diet-Induced Obesity and Regulates Adipogenesis through AMPK

**DOI:** 10.1155/2013/423741

**Published:** 2013-09-19

**Authors:** Hye-Lin Kim, Yong-Deok Jeon, Jinbong Park, Hong-Kun Rim, Mi-Young Jeong, Hara Lim, Seong-Gyu Ko, Hyeung-Jin Jang, Byung-Cheol Lee, Kyung-Tae Lee, Kang-Min Lee, Hyejung Lee, Sung-Hoon Kim, Su-Jin Kim, Seung-Heon Hong, Jae-Young Um

**Affiliations:** ^1^College of Korean Medicine, Institute of Korean Medicine, Kyung Hee University, Hoegi-Dong, Dongdaemun-Gu, Seoul 130-701, Republic of Korea; ^2^Department of Oriental Pharmacy, College of Pharmacy, Wonkwang-Oriental Medicines Research Institute, Wonkwang University, Jeonbuk 570-749, Republic of Korea; ^3^Department of Pharmaceutical Biochemistry, College of Pharmacy, Kyung Hee University, Hoegi-Dong, Dongdaemun-Gu, Seoul 130-701, Republic of Korea; ^4^Department of Molecular Biology, College of Natural Science, Chonbuk National University, Jeollabuk-do 561-756, Republic of Korea; ^5^Department of Cosmeceutical Science, Daegu Haany University, Yugok-dong, Kyungsan 712-715, Republic of Korea

## Abstract

Obesity is a metabolic disorder characterized by chronic inflammation and dyslipidemia and is a strong predictor for the development of hypertension, diabetes mellitus, and cardiovascular disease. This study examined the antiobesity effect of an ethanol extract of Corni Fructus containing formulation (CDAP), which is a combination of four natural components: Corni Fructus, Dioscoreae Rhizoma, Aurantii Fructus Immaturus, and Platycodonis Radix. The cellular lipid content in 3T3-L1 adipocytes was assessed by Oil Red O staining. Expressions of peroxisome proliferator-activated receptor-**γ** (PPAR-**γ**), CCAAT/enhancer-binding protein-**α** (C/EBP-**α**), and lipin-1 were determined by real-time RT-PCR. Western blot was used to determine the protein levels of PPAR-**γ**, C/EBP-**α**, and AMP-activated protein kinase-**α** (AMPK-**α**). The CDAP extract suppressed the differentiation of 3T3-L1 adipocytes by downregulating cellular induction of PPAR-**γ**, C/EBP-**α**, and lipin-1. The CDAP extract also significantly upregulated phosphorylation of AMPK-**α**. An *in vivo* study showed that CDAP induced weight loss in mice with high-fat-diet-induced obesity. These results indicate that CDAP has a potent anti-obesity effect due to the inhibition of adipocyte differentiation and adipogenesis.

## 1. Introduction

Obesity is the main cause of metabolic syndrome, which can lead to various complications, including hardening of the arteries and an increased risk of cardiovascular diseases. Therefore, obesity has a large impact on healthcare in both developed and developing countries. Obesity is characterized by excessive fat deposition and is associated with morphological and functional changes in the adipocytes [1]. Accordingly, understanding the mechanisms by which particular herbal medicines affect adipocyte differentiation could help prevent obesity and its associated diseases [2].

Adipose tissue, an important depot for energy storage, regulates energy homeostasis. Excessive increases in the number and size of adipocytes result in obesity and metabolic syndrome. Adipogenesis, a differentiation process of adipocytes, involves changes in gene expression and cellular morphology. Adipocyte hypertrophy results from an excessive accumulation of lipids from intake of excessive energy sources such as a high-fat (HF) diet. Changes in the number of adipocytes result from a complex interplay between proliferation and differentiation of preadipocytes [3]. Therefore, understanding the molecular mechanism of adipocyte differentiation is necessary for the efficient treatment of these diseases. To elucidate the molecular mechanisms of adipogenesis, 3T3-L1 cells have been generally used as an *in vitro* model. Differentiation of 3T3-L1 preadipocytes into mature adipocytes can be induced by upstimulation with 3-isobutyl-1-methylxanthine (IBMX), dexamethasone (Dex), and insulin and promotes the accumulation of large amounts of intracellular lipid droplets in mature adipocytes [4]. During adipogenesis of 3T3-L1 cells, peroxisome proliferator-activated receptor-*γ* (PPAR-*γ*) and CCAAT/enhancer-binding protein-*α* (C/EBP-*α*) play key roles as major transcription factors [5]. Expression of PPAR-*γ*, a transcription factor of the nuclear-receptor superfamily, and C/EBP-*α*, a member of the C/EBP family of basic-leucine zipper class of transcription factors, increased during differentiation of 3T3-L1 cells [6].

AMP-activated protein kinase (AMPK) is a “cellular fuel gauge” and acts to simultaneously shut down ATP-consuming biosynthetic processes and facilitate ATP-producing catabolic processes during periods of metabolic stress, leading to rapid changes in the control of fatty acid metabolism. AMPK stimulation of fatty acid metabolism occurs as a result of AMPK phosphorylation [7]. AMPK, composed of a, b, and g subunits, is a key player in energy homeostasis. When the intracellular AMP/ATP ratio increases because of metabolic stress, AMPK is phosphorylated. Subsequently, downstream target molecules are activated, promoting catabolism. When the intracellular AMP/ATP ratio decreases, AMPK increases anabolism. AMPK is associated with adipocyte differentiation via AMPK activation in 3T3-L1 adipocytes [8, 9]. Lipin is also a central regulator of adipose tissue development. Mammalian lipin proteins have been shown to control gene expression and to enzymatically convert phosphatidate to diacylglycerol, an essential precursor in triacylglycerol and phospholipid synthesis [10]. Previous studies established that lipin-1 is required at an early step in adipocyte differentiation for induction of the adipogenic gene transcription program, including the key regulator PPAR-*γ* [11]. 

In this study, we investigated the effect of CDAP, a modified prescription of “Supungsunki-hwan.” This frequently prescribed Korean traditional medicine for the treatment of obesity was tested on humans as well as on an animal model subjected to HF diet-induced obesity [12, 13]. The CDAP is a combination of herbal extracts (i.e., Corni Fructus, Dioscoreae Rhizoma, Aurantii Fructus Immaturus, and Platycodonis Radix) at a ratio of 1 : 1 : 1 : 1. This new study investigated the effects of CDAP on the adipocyte differentiation process at the molecular level in 3T3-L1 cells and the effects of dietary CDAP on body weight changes, including physiological and metabolic variables, in HF diet C57BL/6 mice.

## 2. Materials and Methods

### 2.1. Preparation of CDAP

The oriental, medicinal, and herbal mixture used in this experiment (CDAP) is approved as a food ingredient. The herbal sample was prepared as described previously [13]. Briefly, 1 kg each of Corni Fructus, Dioscoreae Rhizoma, Aurantii Fructus Immaturus, and Platycodonis Radix was extracted with 80% ethanol (80% EtOH) for 2 h and 20 min using a heating mantle. The solvents were filtered and evaporated under reduced pressure (Rotary evaporator Model NE-1, Japan) and the remnant then freeze-dried (Freeze dryer FD-1, Japan) at −56°C and 9 mm Torr to acquire extracts of each herbal sample.

### 2.2. Animals and Diets

Male 4-week-old C57BL/6J mice were purchased from Daehan Biolink Co. (Eumsung, Korea) and maintained for one week prior to experiments. All animals were maintained in a C57Bl/6J background, on a 12 h light-dark cycle in a pathogen-free animal facility. Mice were provided with a laboratory diet and water *ad libitum*. All experimental protocols involving the use of animals were conducted in accordance with National Institutes of Health (NIH) guidelines. To induce obesity, the mice were fed a HF diet (Rodent Diet D12492, Research Diet, New Brunswick, NJ, USA) consisting of 60% fat in accordance with previously published reports [14]. Normal mice were fed a commercial standard chow diet (CJ Feed Co., Ltd., Seoul, Republic of Korea). Experimental mice were fed the HF diet for four weeks before administration of CDAP or Slinti, a green tea extract used as a positive control (Myungmoon Pharm. Co., LTD., Seoul, Republic of Korea). The mice were randomly divided into four groups (*n* = 5 per group) that were fed either a normal diet (ND), a HF diet, a HF diet plus CDAP, or a HF diet plus Slinti, for 16 weeks. Body weight and food intake were measured three times per week.

### 2.3. Cell Culture and Differentiation

3T3-L1 mouse embryo fibroblasts were obtained from the American Type Culture Collection (Rockwill, MD, USA). Cells were grown in DMEM plus 10% calf serum and plated for final differentiation in DMEM plus 10% FBS with 100 units/mL of penicillin-streptomycin solution at 37°C, in 5% CO_2_, at 95% humidity until confluence. Two days after confluence (Day 0), the cells were stimulated to differentiate with differentiation inducers (1 *μ*M dexamethasone, 500 *μ*M 3-isobutyl-1-methylxanthine, and 1 *μ*g/mL insulin, MDI) that were added to DMEM containing 10% FBS for two days (Day 2). Preadipocytes were then cultured in DMEM, 10% FBS supplemented with 1 *μ*g/mL insulin for another two days (Day 4), followed by culturing with 10% FBS/DMEM medium for additional two days (Day 6), at which time more than 90% of cells were mature adipocytes with accumulated fat droplets. On Day 2, CDAP was prepared in a differentiation medium at concentrations of 10 *μ*g/mL, 50 *μ*g/mL, and 100 *μ*g/mL.

### 2.4. MTS Cell Viability Assay

On 96-well plates, the 3T3-L1 preadipocytes were seeded (2 × 10^4^ cells/well) and incubated in 10% FBS/DMEM medium for 24 h. Then, the cells were incubated in 10% FBS/DMEM medium containing ethanol extract of CDAP for an additional 48 h. Cell viability was monitored using the Cell Proliferation MTS Kit (Promega Corporation, Madison, WI, USA) as recommended by the manufacturer. Prior to measuring viability, treatment media were removed and replaced with 200 *μ*L fresh 10% FBS/DMEM medium and 10 *μ*L 3-(4,5-dimethylthiazol-2-yl)-5-(3-carboxymethoxyphenyl)-2-(4-sulfophenyl)-2H-tetrazolium (MTS) solution. Cells were then returned to the incubator for 4 h. The absorbance was measured at 490 nm in a VERSAmax microplate reader (Molecular Devices, Sunnyvale, CA, USA) to determine the formazan concentration, which is proportional to the number of live cells.

### 2.5. Oil Red O Staining

Intracellular lipid accumulation was measured using Oil Red O. The Oil Red O working solution was prepared as described by Ramirez-Zacarias et al. [15]. The 3T3-L1 cells were fixed with 10% formalin and then stained for 1 h with filtered solution of 60% Oil Red O in 100% aqueous 2-isopropanol. To quantify the intracellular lipids, the stained lipid droplets were dissolved in isopropanol (3 mL/well). The extracted dye was transferred into a 96-well plate and absorbance read with a VERSAmax microplate reader (Molecular Devices, Sunnyvale, CA, USA) at 500 nm.

### 2.6. RNA Extraction and Real-Time RT-PCR

Total RNA was extracted from the cells using a GeneAll^R^ RiboEx Total RNA extraction kit (GeneAll Biotechnology, Seoul, Republic of Korea) according to the manufacturer's instructions. From 2 *μ*g of RNA, cDNA was reverse-transcribed using a Power cDNA synthesis kit (iNtRON Biotechnology, Seongnam, Kyunggi, Republic of Korea). The primers PPAR*γ*, C/EBP*α*, and glyceraldehyde-3-phosphate dehydrogenase (GAPDH) were used for real-time PCR analysis. Real-time PCR was performed with SYBR Green Power Master Mix (Applied Biosystems, Foster City, CA, USA) using a Step One Plus Real-Time PCR System (Applied Biosystems, Foster City, CA, USA). The target cDNA was amplified using the sense and antisense primers described in Table 1.

### 2.7. Western Blot Analysis

Cultured and differentiated cells were harvested using a cell scraper and then lysed with ice-cold RIPA buffer. The total cell lysates were then centrifuged at 13,000 rpm for 20 min at 4°C to remove the insoluble materials. Next, the total concentration of extracted proteins was determined using the method of Bradford [16]. Western blottings were performed with polyclonal rabbit antibodies against PPAR-*γ* (Cell Signaling Technology, Beverly, MA, USA), C/EBP-*α* (Santa Cruz Biotechnology, Santa Cruz, CA, USA), Lipin-1 (Cell Signaling Technology, Beverly, MA, USA), AMPK-*α* (Cell Signaling Technology, Beverly, MA, USA), p-AMPK-*α* (Cell Signaling Technology, Beverly, MA, USA), and polyclonal mouse antibodies against GAPDH (Santa Cruz Biotechnology, Santa Cruz, CA, USA). The blots were subsequently incubated with horseradish peroxidase (HRP)-conjugated affinipure Goat anti-rabbit IgG (Jackson Immunoresearch Laboratory, USA) or HRP-conjugated affinipure Goat anti-mouse IgG (Jackson Immunoresearch Laboratory, USA). Polyvinylidene difluoride (PVDF) membrane was purchased from Millipore, and the protein assay reagent was obtained from Bio-Rad (Bio-Rad Laboratories, Hercules, CA, USA).

### 2.8. Serum Analysis

All mice were made to fast for 3 h prior to being sacrificed. Plasma was separated immediately after blood sampling by centrifugation at 4,000 ×g for 30 min. The serum was stored at −70°C until being used for assays. High-density lipoprotein (HDL-C) cholesterol, low-density lipoprotein (LDL-C) cholesterol, total cholesterol (TC), and fructosamine were assayed at the Seoul Medical Science Institute (Seoul Clinical Laboratories, Seoul, Republic of Korea). 

### 2.9. Instrumentation

The HPLC was equipped with a vacuum degasser, a quaternary pump, and an automatic sample injection system. Chromatographic separation was performed on a Nucleosil C 18 (150 × 4.6 mm, 5 *μ*m, Teknokroma, Barcelona, Spain). Samples were separated using acetonitrile and phosphate buffer (50 + 50 v/v), pH 5.5, as the mobile phase at a flow rate of 1.0 mL/min at ambient temperature. Initial elution was performed by acetonitrile-aqueous ammonium acetate 20 : 80 (v/v). After 10 min, the linear gradient reached 60% acetonitrile. 

### 2.10. Statistical Analysis

All data, expressed as mean ± standard deviation, were processed statistically using the software SPSS 11.5 for Windows. Values with *P < 0.05 were considered to indicate statistical significance.

## 3. Results

### 3.1. Effects of CDAP on Cytotoxicity and Inhibition of Adipogenesis in 3T3-L1 Adipocytes

**Figure 1:**
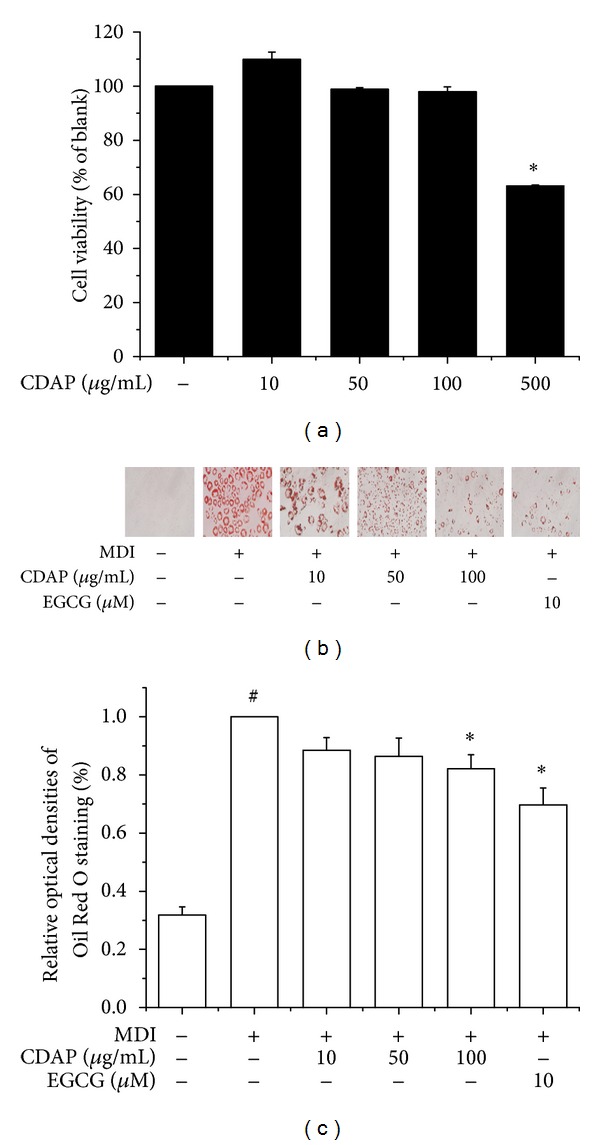
Effect of CDAP on cell viability and lipid accumulation in 3T3-L1 cells. (a) Cells were incubated with CDAP at the indicated concentration for 48 h. Cell viability was assessed by MTS assay. (b) Postconfluent 3T3-L1 cells were differentiated in the absence or in the presence of CDAP (0, 10, 50, and 100 *μ*g/mL) for 6 days. Lipid droplets were measured by Oil Red O staining. EGCG was used as a positive control. All values are mean ± S.D. ^#^ *P* < 0.05 versus undifferentiated control cells; *P < 0.05 versus differentiated control cells.

To determine the cytotoxicity of CDAP, 3T3-L1 cells were treated with various concentrations (10–500 *μ*g/mL) of CDAP and the cell viability measured by the MTS assay. As shown in Figure 1(a), treatment with 10–100 *μ*g/mL of CDAP did not cause significant cytotoxic effects on 3T3-L1 cells. Due to this result, further treatments proceeded at the concentrations of 10, 50, and 100 *μ*g/mL. Next, to investigate the effects of CDAP on preadipocyte differentiation, the lipid accumulation was measured by an Oil Red O staining assay. As shown in Figure 1(b), 100 *μ*g/mL of CDAP suppressed lipid accumulation in 3T3-L1 adipocytes at levels that were statistically significant (*P* < 0.05), suggesting that CDAP inhibits adipogenesis in 3T3-L1 cells. Epigallocatechin gallate (EGCG) was used as a positive control. 

### 3.2. Effect of CDAP on the Expression of PPAR*γ*, C/EBP*α*, and Lipin-1 in 3T3-L1 Adipocytes

**Figure 2:**
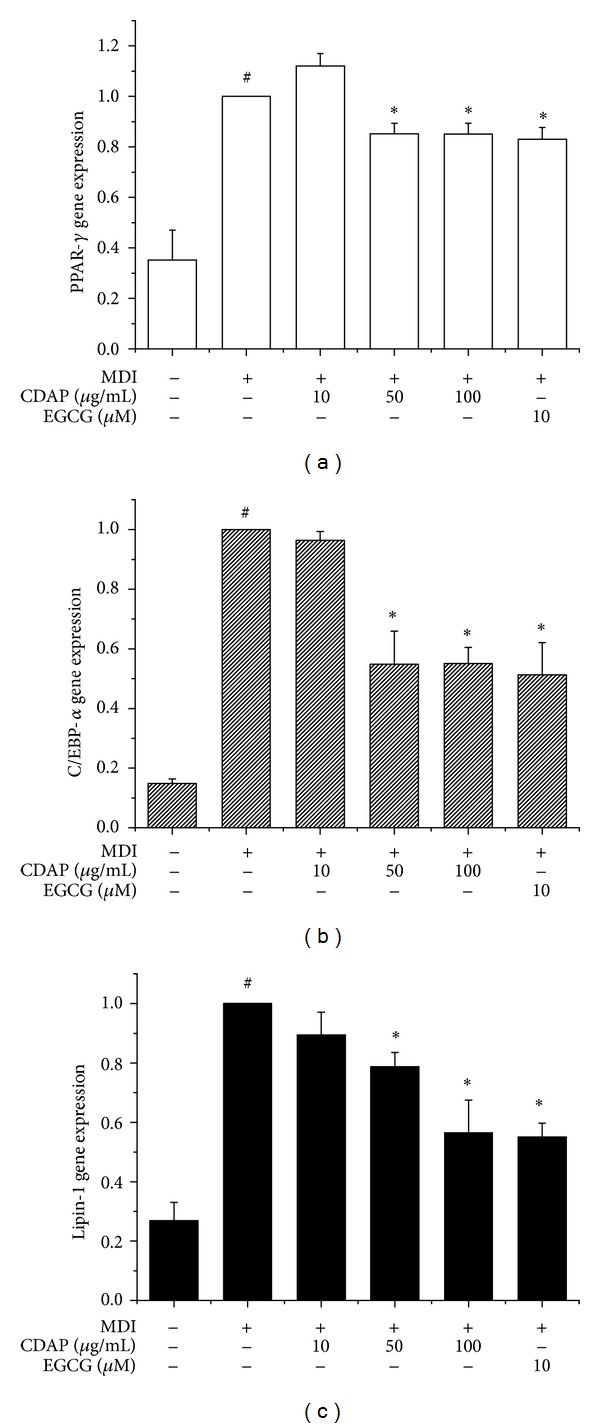
Effect of CDAP on the expression of transcription factors and adipocyte-specific genes in differentiation of 3T3-L1 cells. Postconfluent 3T3-L1 cells were differentiated in the absence or presence of CDAP (0, 10, 50, and 100 *μ*g/mL) for 6 days. The mRNA of PPAR-*γ* (a), C/EBP-*α* (b), and lipin-1 (c) was analyzed by real-time RT-PCR. Results were expressed relative to untreated cells after normalization to GAPDH mRNA. EGCG was used as a positive control. Values are mean ± S.D. of data from three separate experiments; each experiment was performed in triplicate. ^#^ *P* < 0.05 versus undifferentiated control cells; *P < 0.05 versus differentiated control cells.

**Figure 3:**
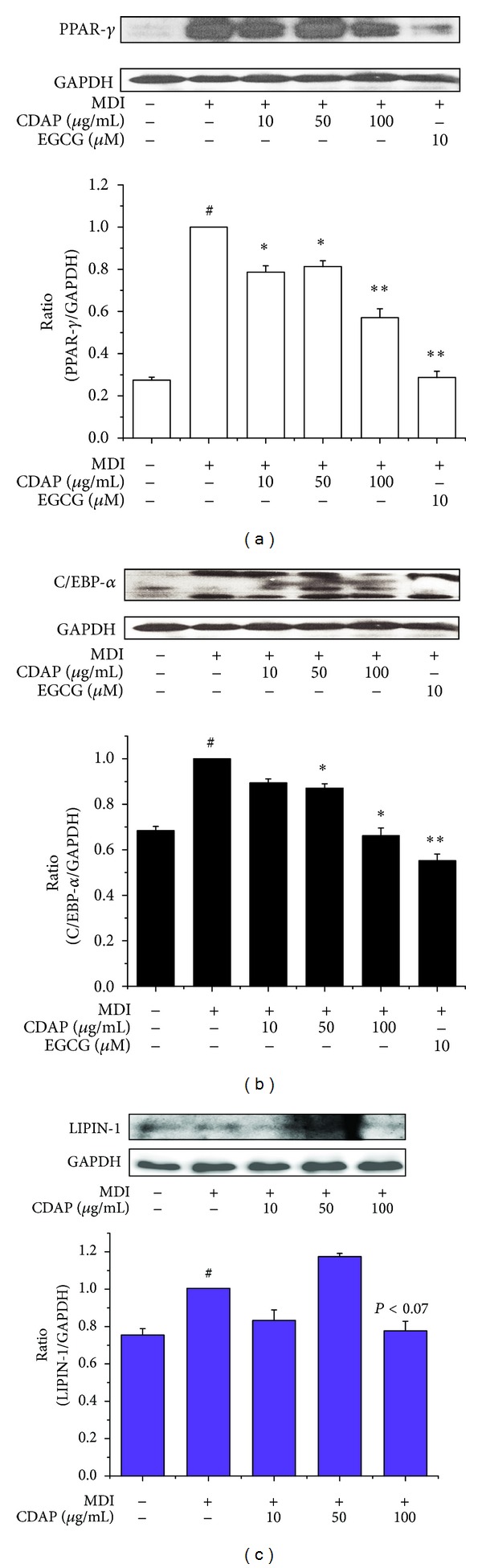
Effect of CDAP on the protein expressions of transcription factors in differentiation of 3T3-L1 cells. Postconfluent 3T3-L1 cells were differentiated in the absence or presence of CDAP (0, 10, 50, and 100 *μ*g/mL) for 6 days. PPAR*γ* (a), C/EBP*α* (b), and lipin-1 (c) protein expressions were analyzed by western blot analysis. EGCG was used as a positive control. Values are mean ± S.D. of data from three separate experiments; each experiment was performed in triplicate. ^#^ *P* < 0.05 versus undifferentiated control cells; *P < 0.05 and **P < 0.01 versus differentiated control cells.

Adipocyte differentiation accompanies the changes in expression of various adipogenic and lipogenic genes [17]. PPAR*γ* and C/EBP*α* are well recognized adipogenic genes known to have roles in the early stage of adipogenesis [18]. Lipin-1 is also required at an early step in adipocyte differentiation for induction of the adipogenic gene transcription [11]. To investigate the antiadipogenic mechanism, the effect of CDAP on mRNA expression levels of PPAR*γ*, C/EBP*α*, and lipin-1 were examined. Fully differentiated 3T3-L1 cells were exposed for 48 h to concentrations of 10, 50, and 100 *μ*g/mL of CDAP. Expressions of both adipogenic genes PPAR*γ* and C/EBP*α* were significantly decreased by CDAP (Figures 2(a) and 2(b)). In addition, CDAP significantly suppressed the expression of lipin-1 in a dose-dependent manner (Figure 2(c)). We also demonstrated that CDAP treatment resulted in a dose-dependent suppression of PPAR*γ*, C/EBP*α*, and lipin-1 at the protein level (Figures 3(a), 3(b), and 3(c)). EGCG was used as a positive control. 

### 3.3. The Effect of CDAP on the Expression and Phosphorylation of AMPK

**Figure 4:**
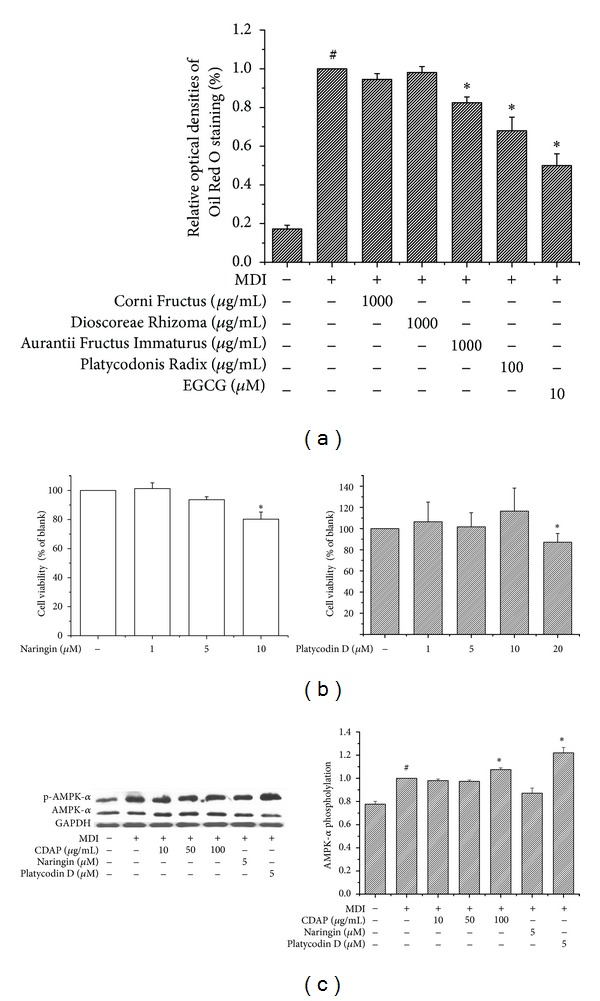
Effect of CDAP and its constituents on phosphorylation of AMPK during 3T3-L1 differentiation. Postconfluent 3T3-L1 cells were differentiated in the presence or absence of Corni Fructus, Dioscoreae Rhizoma, Aurantii Fructus Immaturus, and Platycodonis Radix for 6 days. (a) Lipid accumulation was measured by an Oil Red O staining assay. (b) The cytotoxicity of each constituent, naringin and platycodin D, which are more effective herb's constituents on lipid accumulation, was examined using an MTS assay. (c) Effect of CDAP on AMPK activation was compared with naringin and platycodin D, a major constituent of Aurantii Fructus Immaturus and Platycodonis Radix, respectively. Protein levels of phosphorylated AMPK (pAMPK) and total AMPK were determined by western blot analysis. The protein expression differences are normalized to AMPK-*α*. Values are mean ± S.D. of data from three separate experiments; each experiment was performed in triplicate. ^#^ *P* < 0.05 versus undifferentiated control cells; *P < 0.05 versus differentiated control cells.

To investigate whether AMPK, a key player in energy homeostasis [19], is activated by CDAP during 3T3-L1 differentiation, the level of phosphorylated AMPK*α* was analyzed and compared with the total level of AMPK*α*. When compared with the control group, AMPK*α* phosphorylation increased after treatment with 100 *μ*g/mL of CDAP (Figure 4(c)). A western blot showed that total AMPK*α* was unchanged. In addition, the effect of CDAP on AMPK activation was also compared with naringin and platycodin D, since Aurantii Fructus Immaturus and Platycodonis Radix showed significant antiadipogenic effects on 3T3-L1 differentiation compared to other herb components of CDAP (Figure 4(a)). The cytotoxicity of naringin and platycodin D, major constituents of Aurantii Fructus Immaturus and Platycodonis Radix, respectively, was examined using an MTS assay (Figure 4(b)). The result showed that CDAP had a higher effect on activation of AMPK than that of naringin but not higher than that of platycodin D. 

### 3.4. Effects of CDAP Extract on HF Diet-Induced Obese C57BL/6J Mice

**Figure 5:**
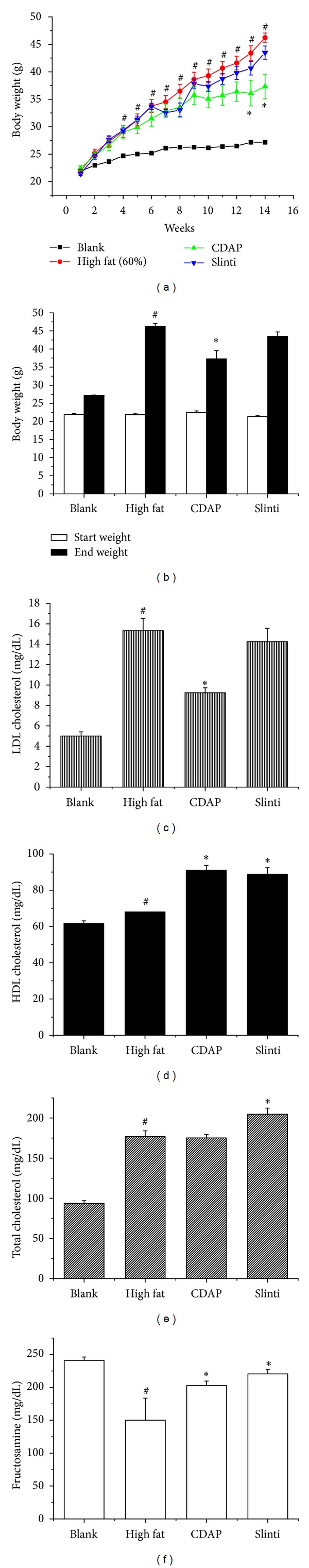
Effects of CDAP in HF diet-induced obesity mice. Mice (*n* = 5 per group) were administrated CDAP extract (100 mg/kg/day) with their HF diet for 16 weeks. Normal diet (blank) fed mice were administrated with vehicle. Slinti (5 mg/kg/day) was administrated as a positive control. Changes in body weight (a), the weight difference between the start weight and end weight of each group (b), LDL cholesterol (c), HDL cholesterol (d), total cholesterol (e), and fructosamine (f) of the mice were measured. All values are mean ± S.D. ^#^ *P* < 0.05 significantly different from blank (normal diet group); *P < 0.05 significantly different from HF diet group (60%).

To examine the reduction of body weight by CDAP, mice were fed HFD for four weeks before administration of either CDAP or Slinti. After inducing obesity, the mice were subdivided into four groups: the normal diet group with vehicle treatment, the 60% HFD group with vehicle treatment, the 60% HFD group with Slinti (5 mg/kg/day) treatment as a positive control, and the 60% HFD group with CDAP (100 mg/kg/day). After inducing obesity with HFD for four weeks, there were significant differences in body weight between the HFD and normal diet groups. The weight gain of the group of mice administered with extract of CDAP (100 mg/kg/day) was significantly (*P* < 0.05) decreased compared with the HD diet group (Figures 5(a) and 5(b)). The level of daily food intake was unchanged, suggesting that the antiobesity effects of CDAP extract were not mediated by a reduction of food and water intake. The changes in the blood plasma parameter are shown in Figures 5(c)–5(f). The CDAP group showed significant decrease in LDL cholesterol compared to the HF diet group. 

### 3.5. Chromatographic Separation

**Figure 6:**
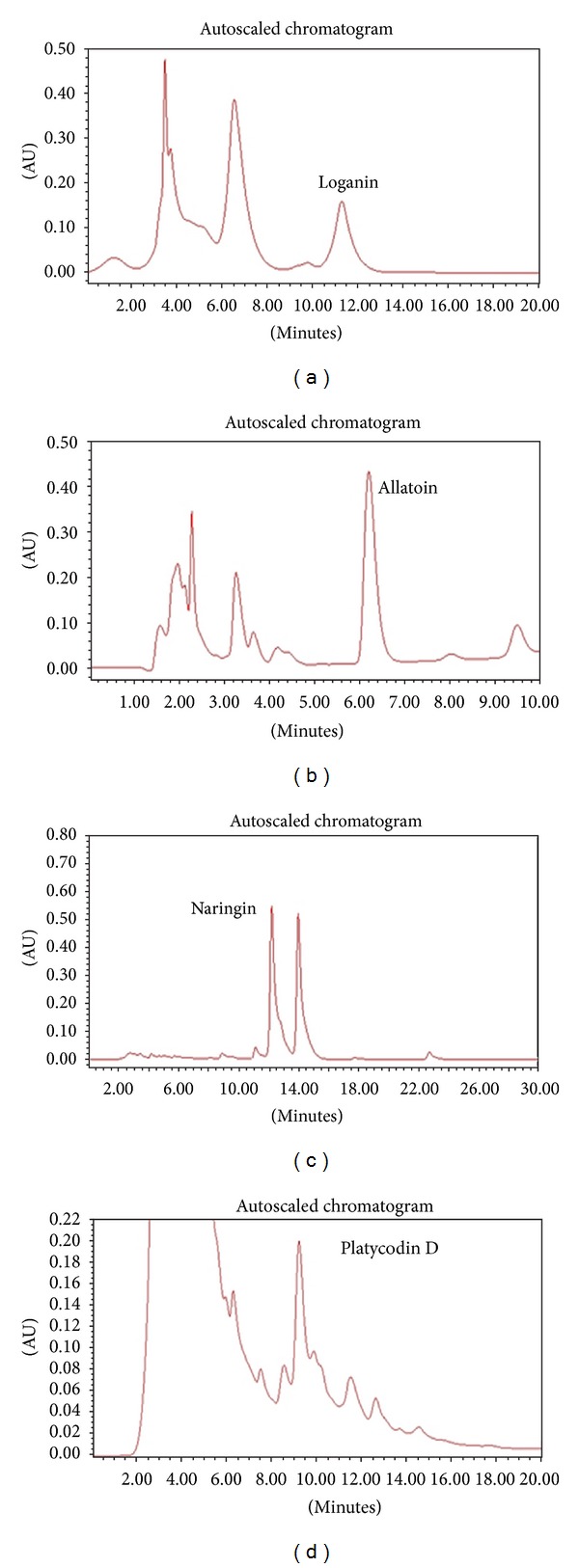
HPLC chromatogram of CDAP. The peaks were assigned based on the isolation of each compound. (a) Corni Fructus: logarnin; (b) Dioscoreae Rhizoma: allantoin; (c) Aurantii Fructus Immaturus: naringin; (d) Platycodonis Radix: platycodin D. The herbs were extracted with 80% ethanol (80% EtOH).

The HPLC chromatogram revealed that loganin (Corni Fructus), allantoin (Dioscoreae Rhizoma), naringin (Aurantii Fructus Immaturus), and platycodin D (Platycodonis Radix) were the major constituents in the organic molecules of the EtOH extract of CDAP (Figure 6).

## 4. Discussion

CDAP is a modified prescription of “Supungsunki-hwan”, a frequently prescribed Korean traditional medicine for the treatment of obesity. The effects of Corni Fructus, Dioscoreae Rhizoma, and Aurantii Fructus Immaturus have been studied for reduction of blood glucose, as well as improving insulin sensitivity [12]. These three herbs are included in the original “Supungsunki-hwan” prescription, which consists of 12 herbal medicines. In addition, it has been reported that platycodin D, a major component of Platycodonis Radix, effectively inhibits triglyceride accumulation in adipocytes [16]. In this study, we examined the anti-obesity effect of ethanol extract of CDAP, a combination of four herbal components (i.e., Corni Fructus, Dioscoreae Rhizoma, Aurantii Fructus Immaturus, and Platycodonis Radix). 

The present study demonstrates the effect of CDAP extract on inhibition of adipocyte differentiation. Our results show that CDAP did not cause significant cytotoxic effects in 3T3-L1 cells and significantly inhibited lipid accumulation and adipocyte differentiation in a concentration-dependent manner. These results indicate that CDAP inhibited adipogenesis during adipocyte differentiation and may have potential anti-obesity effects. As shown in Figure 4, Aurantii Fructus Immaturus and Platycodonis Radix were more effective than other herbs of CDAP to inhibit adipogenesis. In addition, platycodin D, a major component of Platycodonis Radix, was more effective than CDAP to activate AMPK-*α*. However, CDAP reduced the serum levels of aspartate transaminase (AST) and alanine transaminase (ALT), compared to each individual herb (Supplementary Figure S1 available online at http://dx.doi.org/10.1155/2013/423741).

PPAR-*γ*, a transcription factor predominantly expressed in adipose tissue, plays an important role in adipocyte differentiation, lipid storage, and glucose homeostasis [20]. Also, adipogenesis is highly regulated by two primary adipogenic transcription factors, PPAR-*γ* and C/EBPs [21]. Among these, PPAR-*γ* is well known as the master of adipogenic transcription [22]. The expression of PPAR-*γ* leads to induced adipogenesis in mesenchyme stem cells and fibroblasts [23, 24]. PPAR-*γ* is also known to bind to the C/EBP-*α* promoter region that induces the expression of C/EBP-*α* [25]. C/EBP-*α* is a transcription factor of the C/EBP family, basic-leucine zipper class, and is regulated by C/EBP-*β* in adipocyte differentiation [26]. We found that CDAP significantly downregulates PPAR-*γ* and C/EBP-*α* mRNA and the protein levels induced by differentiation medium in 3T3-L1 cells. This could be explained in two ways: CDAP either inhibited PPAR-*γ* and C/EBP-*α* or suppressed the upstream molecules. Lipin-1 is also required in adipocyte differentiation for induction of the adipogenic gene transcription [11]. We found that CDAP can inhibit adipocyte differentiation through suppression of lipin-1. 

AMPK, a central sensor of cellular energy, is a eukaryotic heterotrimeric serine/threonine kinase, and it has emerged as a therapeutic target for metabolic disorders including obesity. The activation of AMPK is essential for the inhibition of 3T3-L1 adipocyte lipogenesis by phytochemicals [27]. The anti-obesity effects of many natural compounds are mediated through the regulation of fat cells. Genistein, EGCG, and capsaicin, in particular, were shown to inhibit adipogenesis by activating AMPK-*α* [28, 29]. To determine whether CDAP inhibits adipocyte differentiation by activating AMPK, the level of AMPK phosphorylation was determined. The results show that the level of AMPK phosphorylation was elevated significantly after CDAP treatment. This result indicates that CDAP inhibited adipocyte differentiation via activation of AMPK. AMPK activator A-769662 inhibits adipocyte differentiation by downregulating PPAR*γ*, C/EBP*α*, FAS, and aP2 [30]. It has been also reported that treatment of 3T3-L1 preadipocytes with an AMPK activator, AICAR, inhibited the differentiation process [31]. Therefore, our result suggests therapeutic potential for using CDAP as an activator of AMPK.

## 5. Conclusion

The results of this study show anti-obesity effects of CDAP both *in vivo* and *in vitro*. CDAP inhibited adipocyte differentiation of 3T3-L1 and reduced weight gain in mice with HF-diet-induced obesity. CDAP significantly decreased lipid accumulation and the expressions of the major adipogenesis factors PPAR-*γ*, C/EBP-*α*, and lipin-1. CDAP also upregulated phosphorylation of AMPK-*α*. These antiobesity effects of CDAP support its potential as a therapeutic substance or as a source of therapeutic substances.

## Supplementary Material

The serum levels of AST and ALT were detected to check any possible internal toxicity in HF diet-induced obese C57BL/6J mice. The CDAP reduced the serum levels of AST and alanine ALT, compared to each individual herb.

## Figures and Tables

**Table 1:** Sequences of oligonucleotide primers (5′ to 3′) for real-time RT-PCR.

Genes	5′ to 3′ oligonucleotide sequences
Mouse PPAR-*γ*	
Sense (forward)	TTT TCA AGG GTG CCA GTT TC
Antisense (reverse)	TTA TTC ATC AGG GAG GCC AG
Mouse C/EBP-*α*	
Sense (forward)	GCC GAG ATA AAG CCA AAC AA
Antisense (reverse)	CCT TGA CCA AGG AGC TCT CA
Mouse lipin-1	
Sense (forward)	CGC CAA AGA ATA ACC TGG AA
Antisense (reverse)	TGA AGA CTC GCT GTG AAT GG
Mouse GAPDH	
Sense (forward)	AAC TTT GGC ATT GTG GAA GG
Antisense (reverse)	GGA TGC AGG GAT GAT GTT CT

PPAR-*γ*: peroxisome proliferator-activated receptor-*γ*; C/EBP-*α*: CCAAT/enhancer-binding protein-*α*; GAPDH: glyceraldehyde-3-phosphate dehydrogenase.
