# Supplementary figures and images for: Corni Fructus Containing Formulation Attenuates Weight Gain in Mice with Diet-Induced Obesity and Regulates Adipogenesis through AMPK
Source: Evid Based Complement Alternat Med. 2013 Sep 19;2013:423741. doi: 10.1155/2013/423741 (PMC3792538; doi:10.1155/2013/423741)

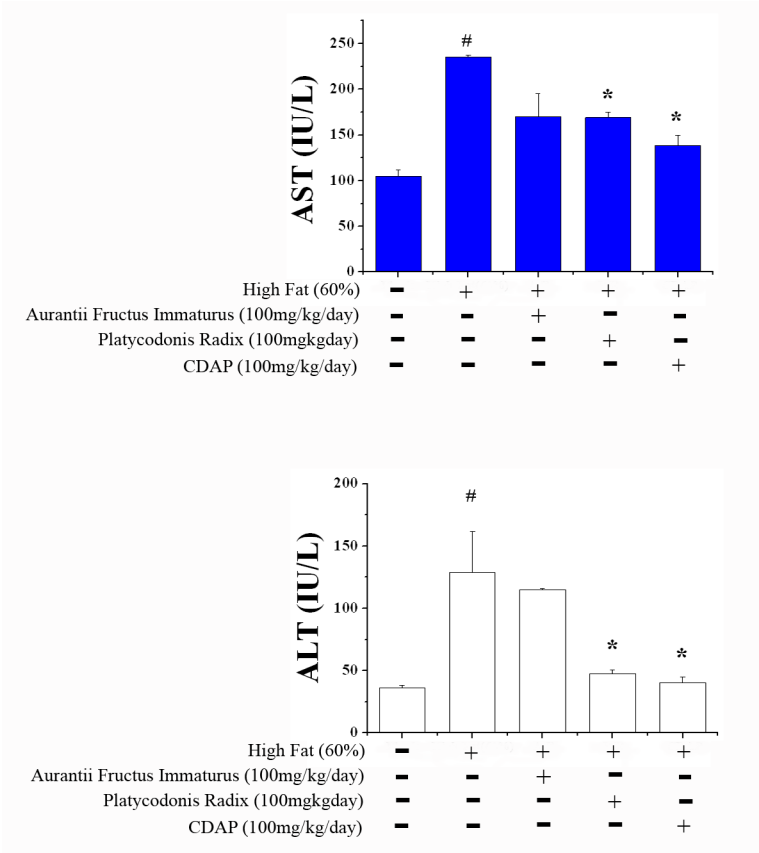

Supplement: Supplementary file 1 — The serum levels of AST and ALT were detected to check any possible internal toxicity in HF diet-induced obese C57BL/6J mice. The CDAP reduced the serum levels of AST and alanine ALT, compared to each individual herb. [file 423741.f1.pdf]
